# Supplementary material for: UTX coordinates TCF1 and STAT3 to control progenitor CD8+ T cell fate in autoimmune diabetes
Source: J Clin Invest. 2025 Dec 16;136(4):e196325. doi: 10.1172/JCI196325 (PMC12904725; doi:10.1172/JCI196325)
Supplement: Supplemental data [file jci-136-196325-s145.pdf]

Supplemental Figures

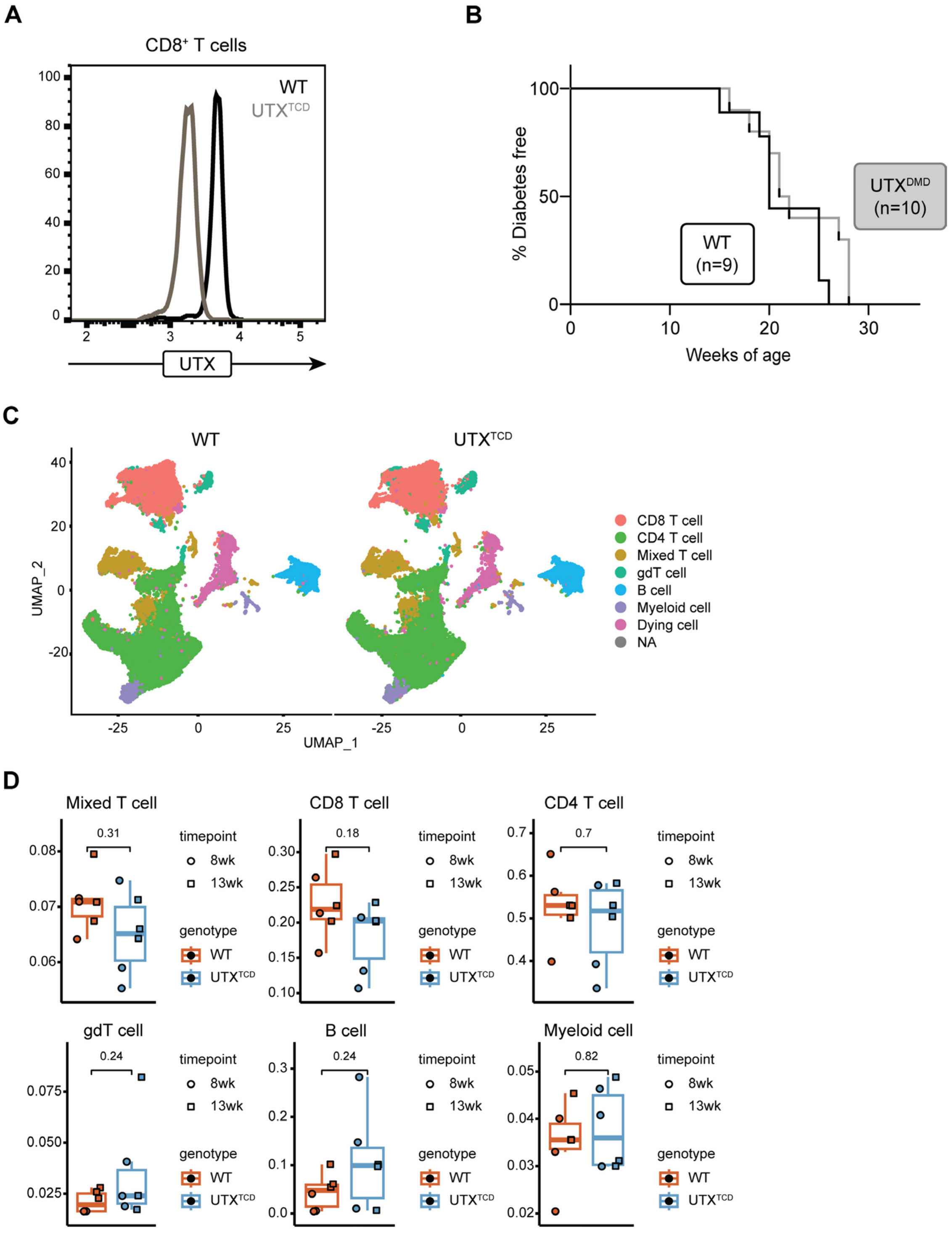

Supplemental Figure 1. Single-cell RNA sequencing of CD45⁺ cell populations in *NOD-UTX^TCD* mice. (A) Flow cytometry analysis on the UTX protein level of WT and UTX^TCD CD8⁺ T cells. **(B)** Diabetes-free incidence curves of *NOD-UTX^DMD* vs. *NOD-WT* female littermates. **(C)** Split UMAP of CD45⁺ cells by genotype. **(D)** Comparison of subset frequencies of CD45⁺ cell subsets. P values, two-sided, unpaired Mann-Whitney test.

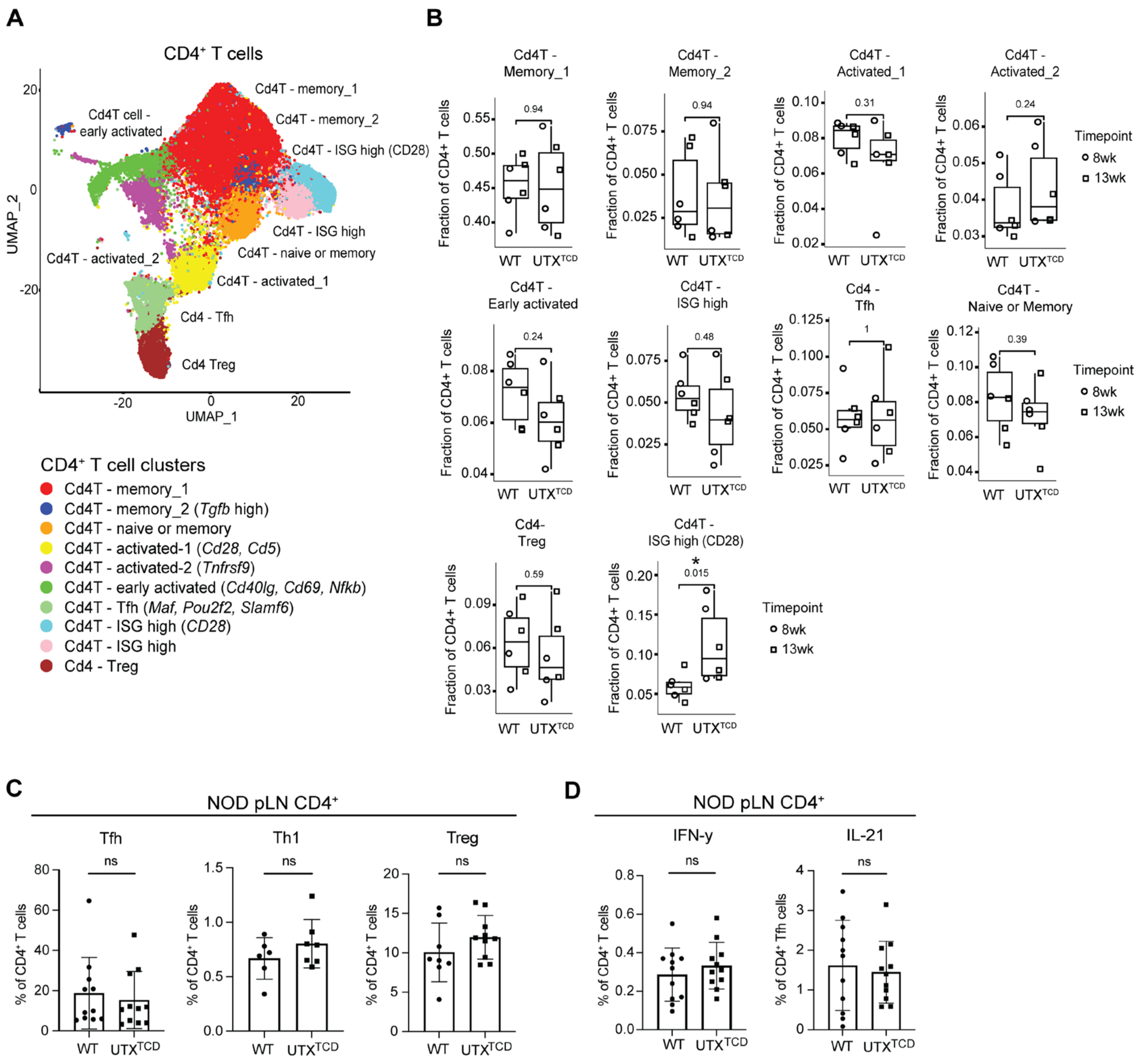

Supplemental Figure 2. Minimal changes in CD4⁺ T cells from *NOD-UTX^TCD* mice. (A) UMAP of CD4⁺ T cells. **(B)** Comparison of subset frequencies of CD4⁺ T cell subsets. P values, two-sided, unpaired Mann-Whitney test. **(C)** Average frequencies of Tfh (CXCR5⁺ PD1⁺), Th1 (T-bet⁺), and Treg (Foxp3⁺) cells among CD4⁺ T cells in pLN of *NOD-WT* and *NOD-UTX^TCD* female littermates. ns= not significant; unpaired Student's t-test. **(D)** Average frequencies of IFN-y⁺ among CD4⁺ T cells and IL-21⁺ among CD4⁺ Tfh cells in pLN of *NOD-WT* and *NOD-UTX^TCD* female littermates. ns = not significant; unpaired Student's t-test.

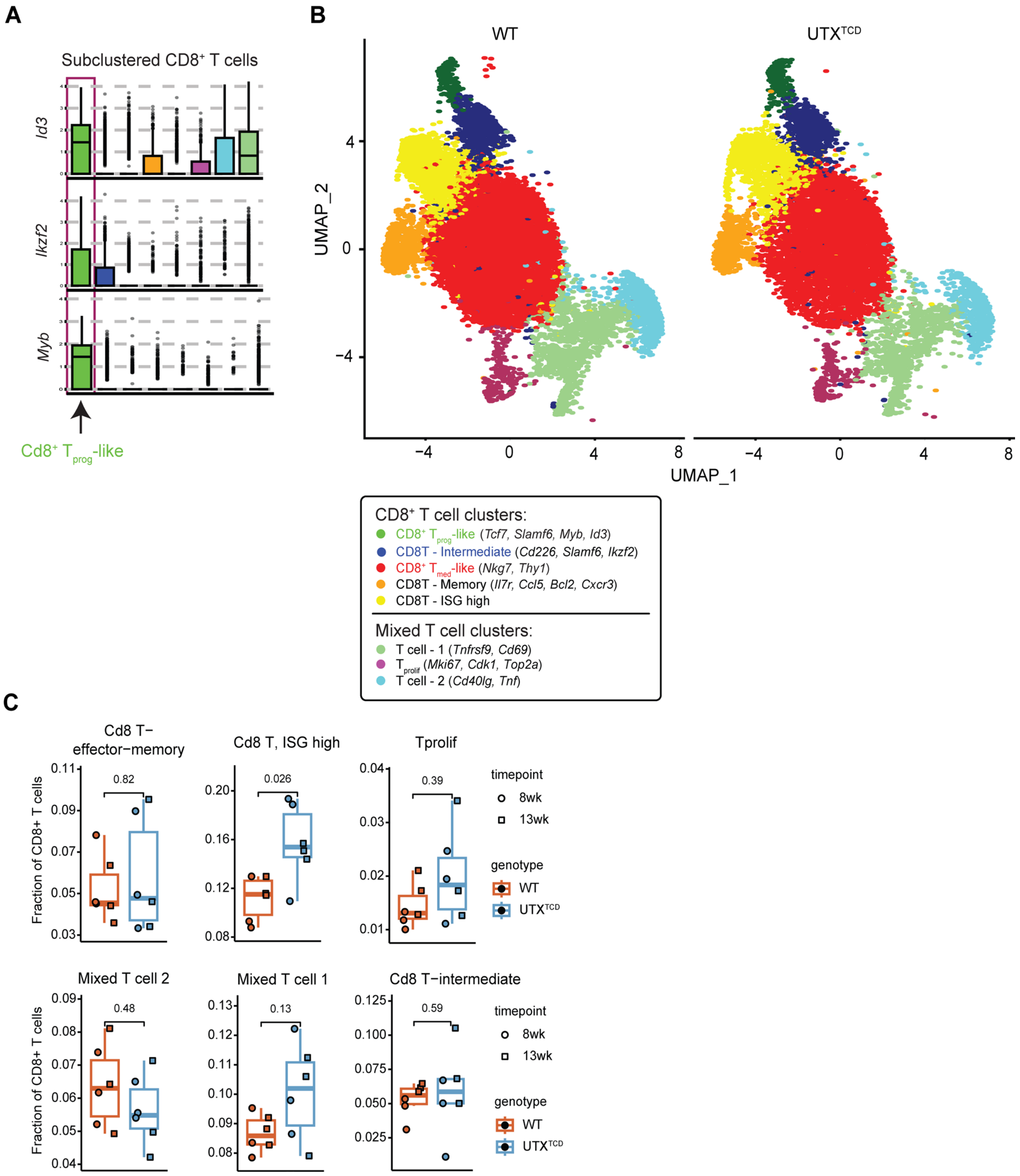

Supplemental Figure 3. CD8⁺ T cells from *NOD-UTX^{TCD}* mice. (A) Expression of key genes (*Id3, Ikzf2,* and *Myb*) across CD8⁺ T cell subclusters. **(B)** Split UMAP of CD8⁺ T cells by genotype. **(C)** Comparison of subset frequencies of CD8⁺ T cell subsets. Subset frequencies for "CD8 Tmed-like" and "CD8 Tprog-like" populations are shown in **Figure 1H**. P values, two-sided, unpaired Mann-Whitney test.

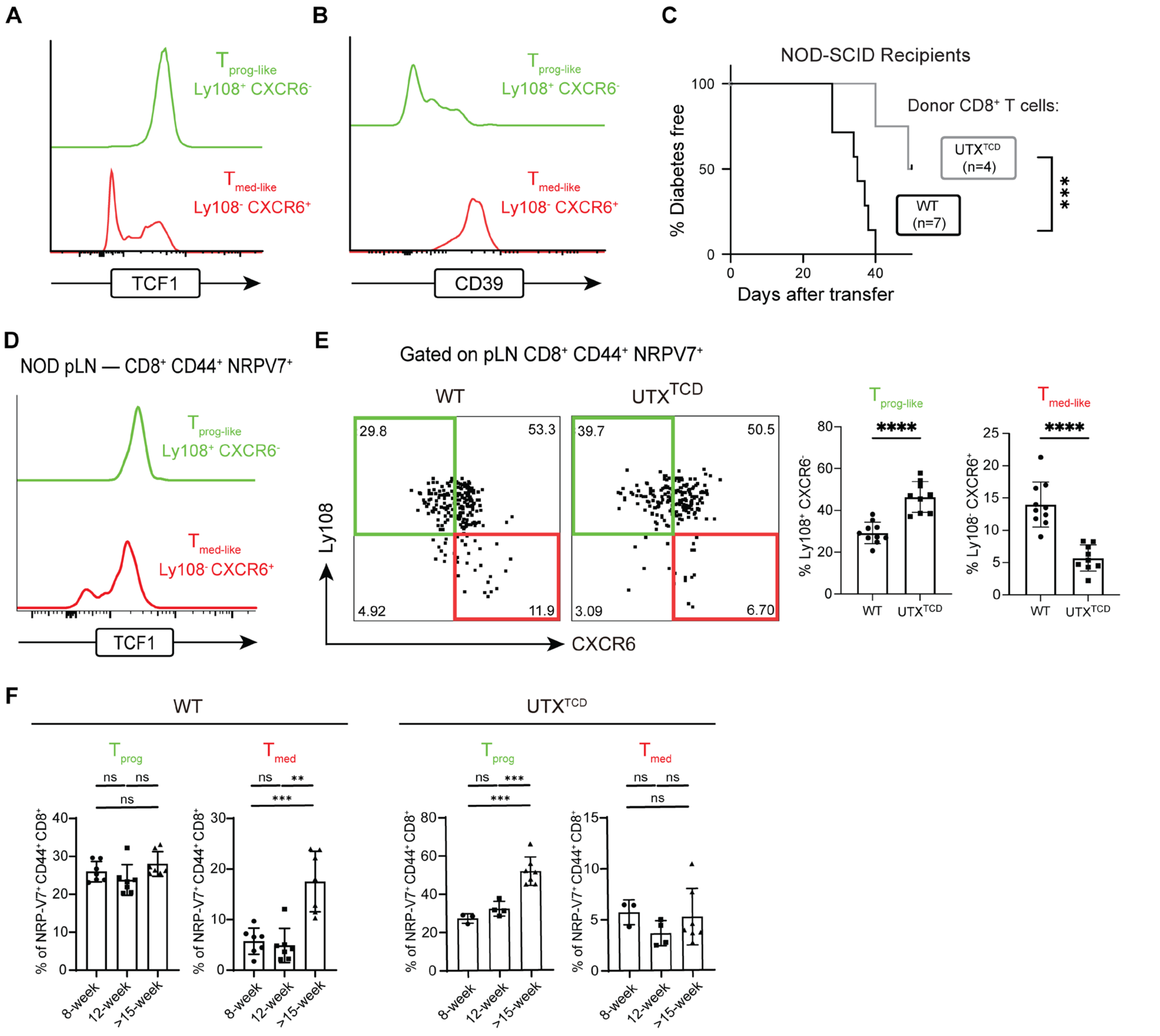

Supplemental Figure 4. Altered CD8^{+} T cell subset distribution in pLN of *NOD-UTX^{TCD}* mice. **(A)** Representative histogram of TCF1 (stem-like marker) and **(B)** CD39 (effector marker) expression in T_{prog}-like vs. T_{med}-like cell populations. **(C)** Diabetes incidence curves of *NOD-SCID* mice recipients transferred with WT CD4^{+} T cells and either *NOD-WT* CD8^{+} T cells or *NOD-UTX^{TCD}* CD8^{+} T cells. ***p<0.001; Log rank test. **(D)** Representative histogram of TCF1 (stem-like marker) in T_{prog}-like (Ly108^{+} CD39^{-}) vs. T_{med}-like (Ly108^{-} CD39^{+}) in pancreatic lymph nodes. **(E)** Representative flow cytometric plot (left) and average frequencies (right) of T_{prog}-like (Ly108^{+} CXCR6^{-}) and T_{med}-like (Ly108^{-} CXCR6^{+}) cells within antigen-experienced, IGRP-specific pLN CD8^{+} T cells (CD8^{+} CD44^{+} NRPV7^{+}) of *NOD-WT* and *NOD-UTX^{TCD}* female littermates (12-16 weeks of age). ***p<0.001; Student's t test. **(F)** The frequency of IGRP^{+} T_{prog} (Ly108^{+} CD39^{-}) and T_{med} (Ly108^{-} CD39^{+}) population in 8-week-old, 12-week-old, and over 15-week-old *NOD-WT* and *NOD-UTX^{TCD}* female mice. ***p<0.001; **p<0.01; unpaired two-way Mann Whitney test.

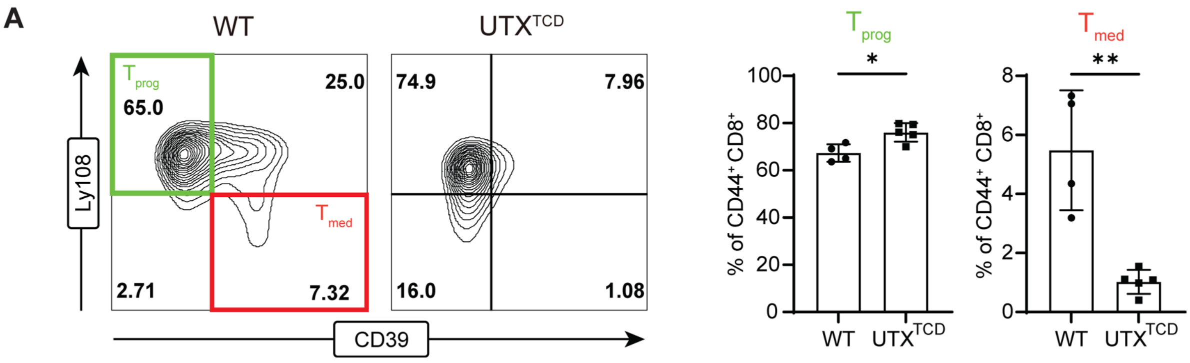

Supplemental Figure 5. The phenotype of CD8$^+$ T cells in the pancreas of *NOD-TCR 8.3 UTX^{TCD}* mice. (A) Representative flow plots (left) and frequencies (right) of T_{prog} and T_{med} in the pancreas of *NOD-TCR 8.3 WT* and *NOD-TCR 8.3 UTX^{TCD}* mice. **p<0.01; *p<0.05; Student's t test.

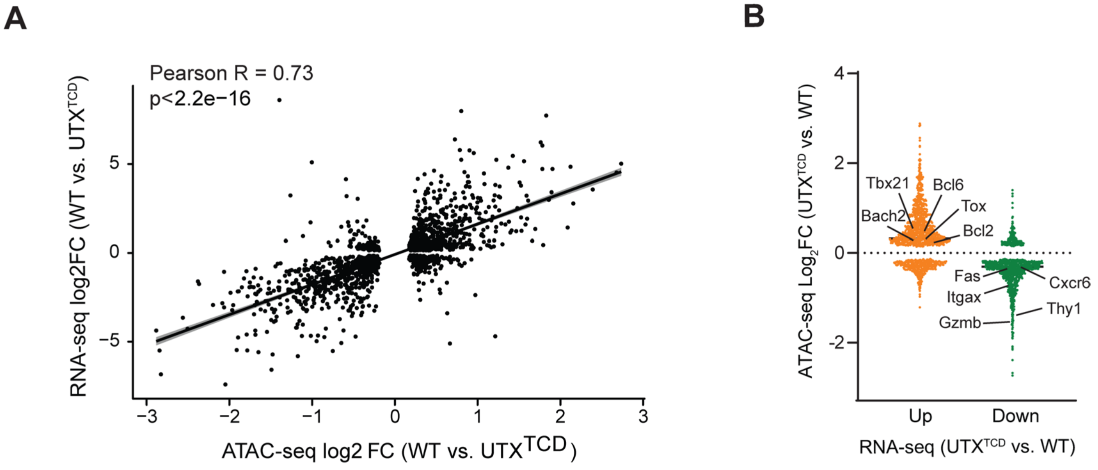

Supplemental Figure 6. Analysis of ATAC-seq data and RNA-seq reveals differentially regulated genes. (A) Correlation between the differential chromatin accessibility and differential gene expression values (in log2FC) in the $CD8^+$ T_{prog} cells of *NOD-TCR 8.3 UTX^{TCD}* compared to *NOD-TCR 8.3 WT* (difference in RNA expression, y-axis; difference in ATAC accessibility, x-axis). R, Pearson correlation coefficient. **(B)** Difference in chromatin accessibility of listed genes (in log2FC) between *NOD-TCR 8.3 UTX^{TCD}* and *NOD-TCR 8.3 WT* $CD8^+$ T_{prog} cells as measured by ATAC-seq (decreased accessibility in *NOD-TCR 8.3 UTX^{TCD}*, log2 FC < -0.5; increased in *NOD-TCR 8.3 UTX^{TCD}*, log2 FC > 0.5).

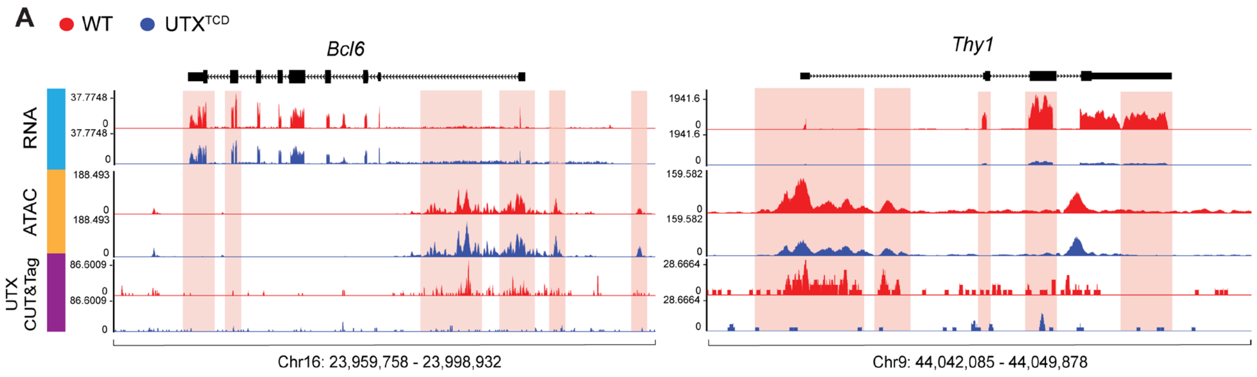

Supplemental Figure 7. UTX deficiency alters chromatin accessibility, gene expression, and associated pathways. (A) Representative gene tracks from UCSC Integrated Genome Browser of UTX CUT&Tag, ATAC-seq, and RNA-seq at Bcl6, and Thy1 gene loci.

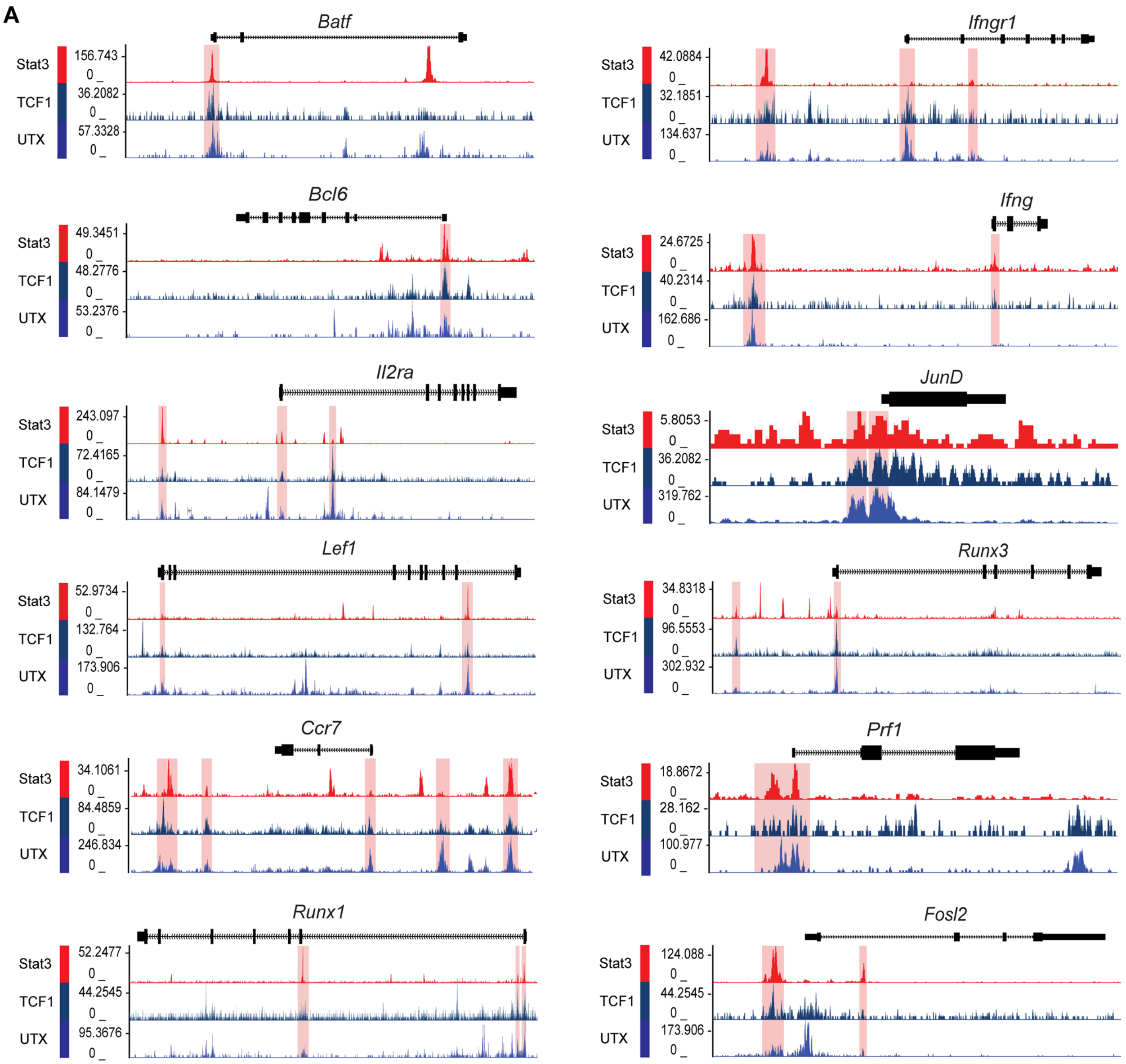

Supplemental Figure 8. Co-occupancy of STAT3, TCF1, and UTX at progenitor and effector genes. (A) Representative gene tracks from the UCSC genome browser to demonstrate the aligned region bound by UTX, TCF1, and Stat3 in progenitor genes (*Batf, Bcl6, Il2ra, Lef1, Ccr7,* and *Runx1*; left) and mediator genes (*Ifngr1, Ifng, JunD, Runx3, Prf1,* and *Fosl2*; right).

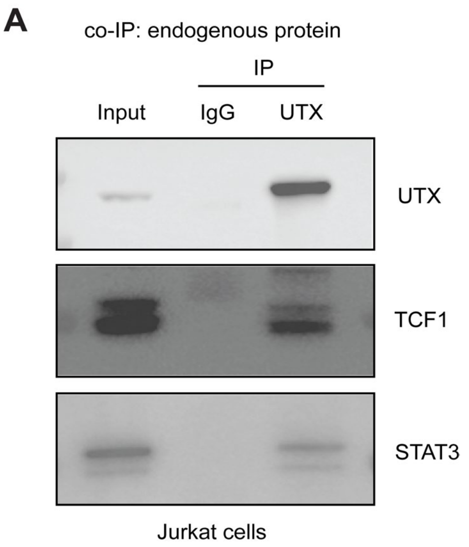

Supplemental Figure 9. UTX interacts with TCF1 and STAT3 in Jurkat T cells. (A) UTX co-immunoprecipitation (co-IP) of endogenous proteins from Jurkat cells. Immunoprecipitants were analyzed by immunoblotting for UTX, TCF1, and STAT3. Input and IgG pulldown controls are shown.

Table S1. Antibodies used for flow cytometry and Western blot experiments.

Brilliant Violet 711™ anti-mouse CD8a Antibody	Biolegend	Cat# 100748; RRID: AB_2562100
APC anti-mouse Ly108 Antibody	Biolegend	Cat# 134610; RRID: AB_2728155
FITC anti-mouse CD186 (CXCR6) Antibody	Biolegend	Cat# 151108; RRID: AB_2572145
APC/Cyanine7 anti-mouse CD3 Antibody	Biolegend	Cat# 100222; RRID: AB_2242784
Alexa Fluor® 700 anti-mouse CD45 Antibody	Biolegend	Cat# 103128; RRID: AB_493715
PE/Dazzle™ 594 anti-mouse/human CD44 Antibody	Biolegend	Cat# 103056; RRID: AB_2564044
BD Horizon™ BUV395 Rat Anti-Mouse CD39	BD Biosciences	Cat# 567264; RRID: AB_2916524
CD279 (PD-1) Monoclonal Antibody (J43), PE, eBioscience™	Thermo Fisher Scientific	Cat# 12-9985-82; RRID: AB_466295
PE/Cyanine7 anti-mouse CD279 (PD-1) Antibody	Biolegend	Cat# 109110; RRID: AB_572017
PE/Cyanine7 anti-mouse CD185 (CXCR5) Antibody	Biolegend	Cat# 145516; RRID: AB_2562210
FITC anti-mouse CD185 (CXCR5) Antibody	Biolegend	Cat# 145520; RRID: AB_2562866
Brilliant Violet 605™ anti-mouse CD4 Antibody	Biolegend	Cat# 100548; RRID: AB_2563054

IL-21 Monoclonal Antibody (mhalx21), PE, eBioscience™	Thermo Fisher Scientific	Cat# 12-7213-82; RRID: AB_1834465
APC anti-mouse IFN-γ Antibody	Biolegend	Cat# 505810; RRID: AB_315404
PE anti-T-bet Antibody	Biolegend	Cat# 644810; RRID: AB_2200542
FOXP3 Monoclonal Antibody (FJK-16s), FITC, eBioscience™	Thermo Fisher Scientific	Cat# 11-5773-82; RRID: AB_465243
Brilliant Violet 711™ anti-human CD8 Antibody	Biolegend	Cat# 344734; RRID: AB_2565243
PE anti-human CD4 Antibody	Biolegend	Cat# 300550; RRID: AB_2564152
PE/Cyanine7 anti-human CD95 (Fas) Antibody	Biolegend	Cat# 305622; RRID: AB_2100369
FITC anti-human CD45RA Antibody	Biolegend	Cat# 304148; RRID: AB_2564157
Brilliant Violet 605™ anti-human CD45RO Antibody	Biolegend	Cat# 304238; RRID: AB_2562153
APC/Cyanine7 anti-human CD197 (CCR7) Antibody	Biolegend	Cat# 353212; RRID: AB_10916390
TCF1/TCF7 (C63D9) Rabbit mAb (PE-Cy7® Conjugate)	Cell Signaling Technology	Cat# 90511S; RRID: AB_3086656
TCF1/TCF7 (C63D9) Rabbit mAb (APC Conjugate)	Cell Signaling Technology	Cat# 37636S; RRID: AB_2922379

KDM6A antibody [N2C1], Internal	GeneTex	Cat# GTX121246; RRID:AB_10722382
InVivoMAb anti-mouse PD-1 (CD279)	BioXCell	Cat# BE0146; RRID:AB_10949053
InVivoMAb mouse IgG2b isotype control	BioXCell	Cat# BE0086; RRID: AB_1107791
Goat Anti-Rabbit IgG H&L (FITC)	abcam	Cat# ab6717; RRID:AB_955238
Tri-Methyl-Histone H3 (Lys27) (C36B11) Rabbit mAb	Cell Signaling Technology	Cat# 9733S; RRID:AB_2616029
UTX (D3Q1I) Rabbit mAb	Cell Signaling Technology	Cat# 9733S; RRID:AB_2721244
DYKDDDDK Tag (D6W5B) Rabbit mAb	Cell Signaling Technology	Cat# 9733S; RRID:AB_2572291
HA-Tag (C29F4) Rabbit mAb	Cell Signaling Technology	Cat# 9733S; RRID:AB_1549585
