# Supplementary figures and images for: UTX coordinates TCF1 and STAT3 to control progenitor CD8+ T cell fate in autoimmune diabetes
Source: J Clin Invest. 2025 Dec 16;136(4):e196325. doi: 10.1172/JCI196325 (PMC12904725; doi:10.1172/JCI196325)

Full unedited gel for Figure 6

Figure 6C

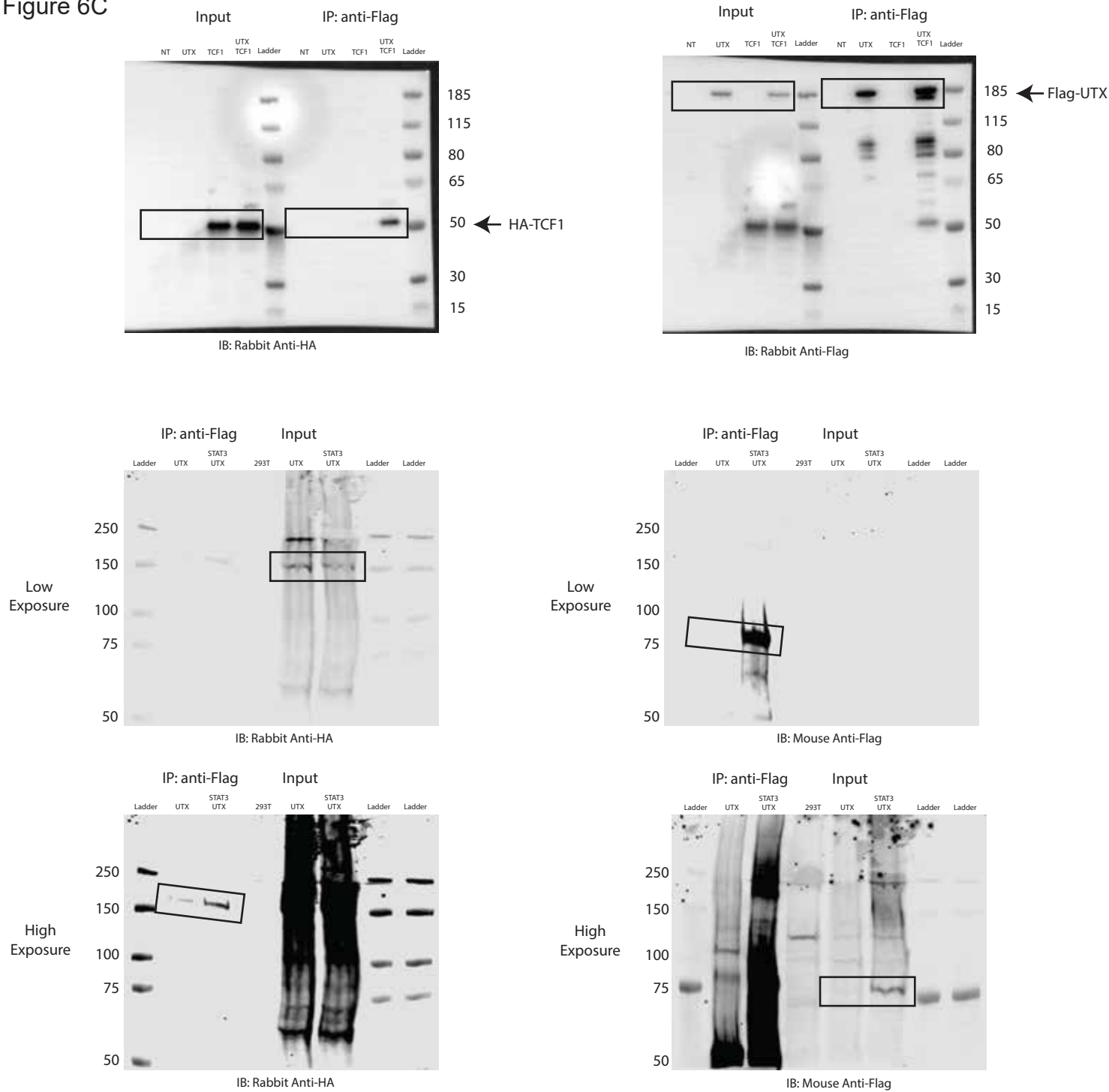

Full unedited gel for Supplementary Figure 9

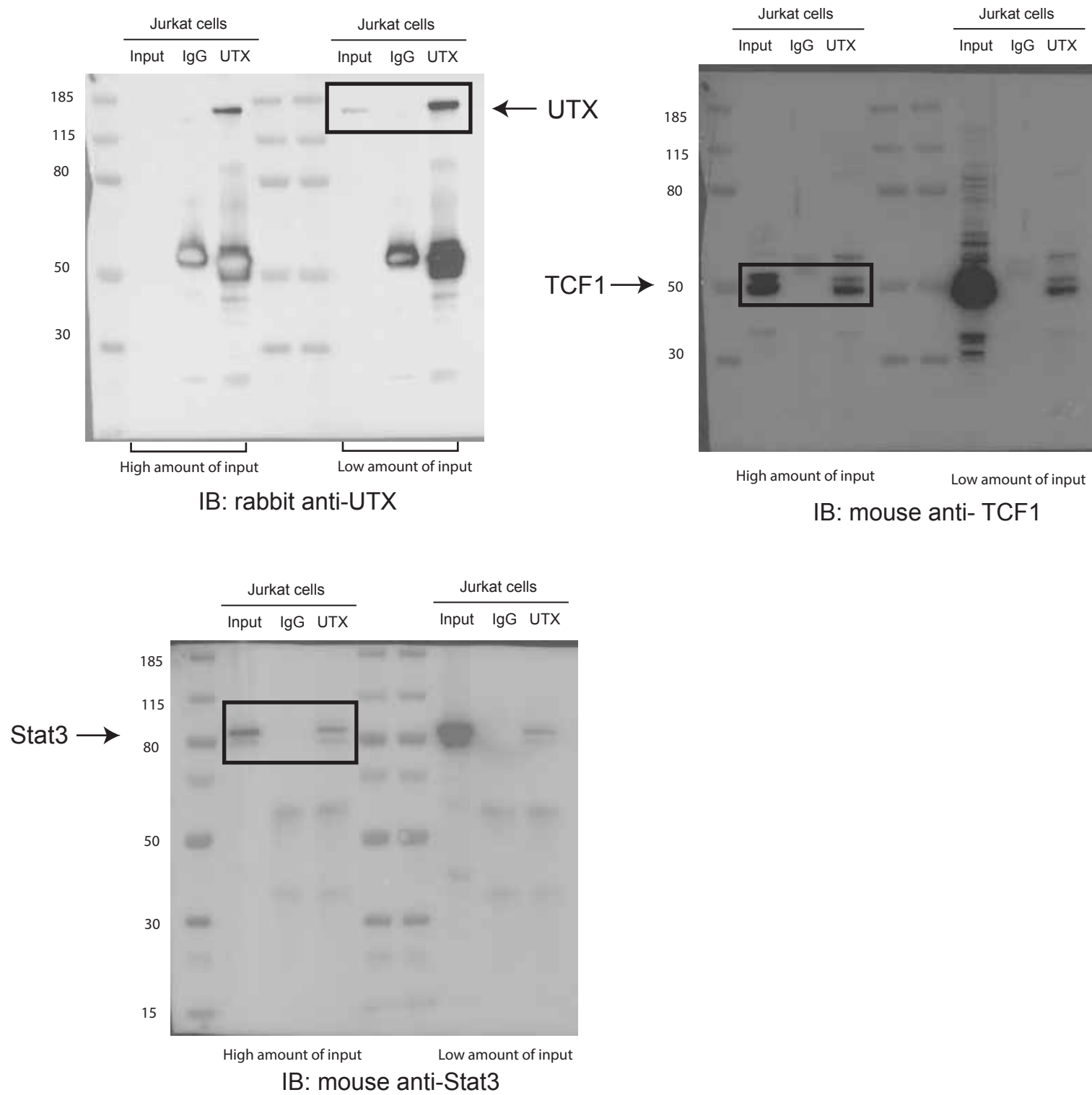

Supplement: Unedited blot and gel images [file jci-136-196325-s151.pdf]
